# Controlling for Participants’ Viewing Distance in Large-Scale, Psychophysical Online Experiments Using a Virtual Chinrest

**DOI:** 10.1038/s41598-019-57204-1

**Published:** 2020-01-22

**Authors:** Qisheng Li, Sung Jun Joo, Jason D. Yeatman, Katharina Reinecke

**Affiliations:** 1 Paul G. Allen School of Computer Science & Engineering, University of Washington, Seattle, WA 98195 USA; 2 Institute for Learning & Brain Sciences, University of Washington, Seattle, WA 98195 USA; 3 Department of Psychology, Pusan National University, Busan, 46241 Republic of Korea; 4 Graduate School of Education, Stanford University, Stanford, CA 94305 USA; 5 Division of Developmental-Behavioral Pediatrics, Stanford University School of Medicine, Stanford, CA 95305 USA

**Keywords:** Psychology, Human behaviour

## Abstract

While online experiments have shown tremendous potential to study larger and more diverse participant samples than is possible in the lab, the uncontrolled online environment has prohibited many types of psychophysical studies due to difficulties controlling the viewing distance and stimulus size. We introduce the Virtual Chinrest, a method that measures a participant’s viewing distance in the web browser by detecting a participant’s blind spot location. This makes it possible to automatically adjust stimulus configurations based on an individual’s viewing distance. We validated the Virtual Chinrest in two laboratory studies in which we varied the viewing distance and display size, showing that our method estimates participants’ viewing distance with an average error of 3.25 cm. We additionally show that by using the Virtual Chinrest we can reliably replicate measures of visual crowding, which depends on a precise calculation of visual angle, in an uncontrolled online environment. An online experiment with 1153 participants further replicated the findings of prior laboratory work, demonstrating how visual crowding increases with eccentricity and extending this finding by showing that young children, older adults and people with dyslexia all exhibit increased visual crowding, compared to adults without dyslexia. Our method provides a promising pathway to web-based psychophysical research requiring controlled stimulus geometry.

## Introduction

Psychophysical methodologies have been extensively applied to study human perception and performance in healthy adults, and to study individual differences across participants and in relation to a variety of clinical conditions. Yet most psychophysical studies are constrained to the laboratory because of the need to rigorously control visual stimulus presentation with the help of a physical chinrest. Given the difficulty of bringing participants into a lab, these studies generally rely on small samples and can risk generalizability to the larger population.

To conduct studies with larger and more diverse samples, researchers have developed and evaluated alternative ways to recruit participants, such as through the online labor market Amazon Mechanical Turk (MTurk) (e.g.^1,2^) or through volunteer-based online experiment platforms such as LabintheWild^3^. Compared to traditional laboratory experiments, such online studies offer faster and more effortless participant recruitment^4–7^ and have resulted in large-scale studies comparing multiple demographic groups, ages, languages, and countries^3,8–11^. A growing body of literature has explored methodologies for conducting a broad range of experiments, and shown that online experiments yield results comparable to those obtained in conventional laboratory settings^1,10,12–20^.

For instance, online experiments have been shown to accurately replicate the findings from behavioral experiments that rely on reaction time measurement^1,14–18^, rapid stimulus presentation^1,12,13^ and learning tasks with complex instructions^1^. De Leeuw and Motz conducted a visual search experiment with interleaved trials implemented in both the Psychophysics Toolbox (in lab) and JavaScript (online) and showed that both software packages were equally sensitive to changes in response times^19^. Similarly, Reimers and Stewart demonstrated that two major ways of running experiments online, using Adobe Flash or JavaScript, can both be used to accurately detect differences in response times despite differences in browser types and system hardware (machines)^20^. Researchers have also investigated if web-based within-subjects experiments studying visual perception can accurately replicate prior laboratory results^2,21^. These online experiments replicated prior laboratory results despite not being able to control for participants’ viewing distance and angle.

To the best of our knowledge, no prior work has investigated whether laboratory results of studies using a physical chinrest can be replicated online for between-subjects experiments in which metrics are being compared across participants, and therefore require tight control of a participants’ viewing distance. To fill this gap, we developed the Virtual Chinrest, a novel method to accurately measure a person’s viewing distance through the web browser. To estimate an individual’s viewing distance, we measure the eccentricity of their blind spot location. We show that our method enables remote, web-based psychophysical experiments of human visual perception by making it possible to automatically adjust stimulus size and location to a participant’s individual viewing distance.

## The Virtual Chinrest

Our approach includes two tasks, first estimating an individual’s screen resolution followed by their viewing distance:

### Screen resolution

**Figure 1:**
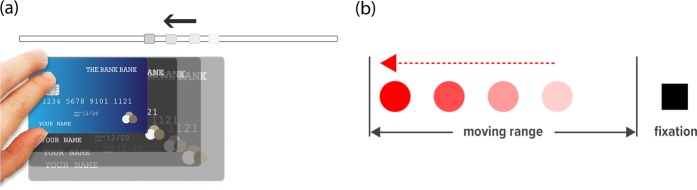
Card Task and Blind Spot Task procedures that are used to calculate the viewing distance using the Virtual Chinrest. (**a**) Card Task: Participants are asked to place a credit card or a card of equal size on the screen, and adjust the slider until the size of the image of the card on the screen matches the real-world card. We can therefore calculate the logical pixel density (LPD) of the display in pixels per inch to estimate distance *s* in Fig. 2. (**b**) Blind Spot Task: Participants are asked to fixate on the static black square with their right eye closed while the red dot repeatedly sweeps from right to left; they are asked to press the spacebar when they perceive the red dot as disappearing. We then calculate the distance between the center of the black square and the center of the red dot when it disappears from the eye sight.

One challenge for conducting psychophysical experiments in the web browser is that the resolution and size of the display are unknown, prohibiting control of the size and location of stimuli presented to participants. To estimate the screen resolution, we calculate the *logical pixel density* (*LPD)* (in pixels per mm) of a display using a card task. We adopted a method that is already commonly used on the internet to help people measure items on the screen: As shown in Fig. 1a, we ask participants to place a real-world object (in our case a credit card or a card of equal size, which are standardized in size and widely available) on a specific place on the screen. Participants can adjust a slider until the size of an image of the object on the screen matches the real-world object. We then calculate the ratio between the card image width in pixels and the physical card width in mm to obtain the LPD in pixel per mm: *LPD* = *cardImageWidth/*85.60 where *cardImageWidth* is the width of the card image in the web browser in pixels after the participant adjusted the slider and 85.60 mm is the width of the card in the real world. Knowing the LPD, we can present online participants with stimuli of a precise size in pixels (on-screen distance) independent of their individual display sizes and resolutions where:

1 $${\rm{LPD}}\,(px/mm)=\frac{\text{On} \mbox{-} \text{screen}\,{\rm{Distance}}\,(px)}{{\rm{Physical}}\,{\rm{Distance}}\,(mm)}$$

We will use this ratio (LPD) to convert between the on-screen distance and physical distance in the following calculation of the viewing distance.

### Viewing distance

**Figure 2:**
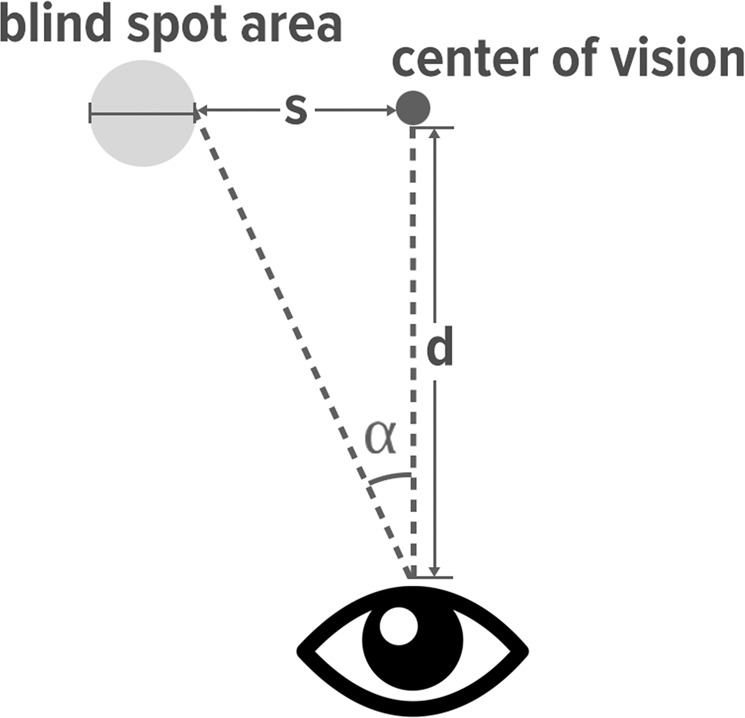
Trigonometric calculation of a participant’s viewing distance using the human eye’s blind spot. Knowing the distance between the center of display and the entry point of the blind spot area (*s*), and given that *α* is always around 13.5°, we can calculate the viewing distance (*d*).

The most critical issue for web-based online psychophysical experiments is how to control stimulus geometry given unknown viewing distance. To tackle this issue, we devised a method in which we leverage the fact that the entry point of the optic nerve on the retina produces a blind spot where the human eye is insensitive to light. The center of the blind spot is located at a relatively consistent angle of α = 15° horizontally (14.33° ± 1.3° in Wang *et al*.^22^, 15.5° ± 1.1° in Rohrschneider^23^, 15.48° ± 0.95° in Safran *et al*.^24^, and 15.52° ± 0.57° in Ehinger *et al*.^25^). Given this, we can calculate an individual’s viewing distance from simple trigonometry, as shown in Fig. 2. More precisely, the LPD obtained from the card task lets us calculate the physical distance *s* by Eq. 1. Once we have detected the blind spot area, we can then calculate the viewing distance *d*.

Inspired by different educational blind spot animations existing online (e.g.^26^), we designed and developed a browser-based blind spot test to estimate the physical distance between one’s blind spot area and the center of display. Participants are asked to fixate on a static black square with their right eye closed while a red dot moves away from fixation (Fig. 1b). The red dot repeatedly sweeps from right to left. At a certain point on the display, the participant will perceive the dot as if it were disappearing. The participant is instructed to press a button when the dot disappears. We then calculate the distance between the center of the black square and the center of the red dot (when it disappears from the eye sight). Instead of using α = 15° as the average horizontal blind spot location as found in previous work^22–25^, we use 13.5° as the average blind spot angle because (1) our method captures the entry point of blind spots (of which the angle should be smaller) instead of the blind spot center, and (2) we calibrated our method by conducting preliminary experiments with a few participants and found that using 13.5° provided us with the most accurate results. The complete formula to calculate the individual viewing distance is

2 $${\rm{Viewing}}\,{\rm{Distance}}\,(d)=\frac{{\rm{Physical}}\,{\rm{Distance}}(s)}{\tan \,(\alpha )}$$

## Results

### Validation experiments in the lab

We conducted two controlled lab experiments to verify that our Virtual Chinrest method is valid and accurate.

#### Exp. 1. Validation of the virtual chinrest with a physical chinrest

The aim of our first experiment was to compare the accuracy of the viewing distance calculated with our Virtual Chinrest method to the viewing distance defined by a physical chinrest. Nineteen participants took part in the experiment with a physical chinrest, fixing their viewing distances at 53.0 cm. The experiment was implemented in JavaScript and run in the web browser; the two tasks of the experiment are schematized in Fig. 1a,b.

To our surprise, despite unavoidable sources of error such as variability of the blind spot location and of the response when the dot disappears, the viewing distance estimates were 53.0 ± 0.69 cm (mean ± standard error of the mean (sem)), which is very accurate given the physical viewing distance of 53.0 cm. The average absolute error was 2.36 cm.

#### Exp. 2. Distance calculation with different display sizes & viewing distances and no physical chinrest

In Exp. 2, we tested the accuracy of the Virtual Chinrest method when systematically changing the display size and participants’ viewing distances. Participants did not use a physical chinrest in this experiment; instead, we controlled for participants’ seating distances (defined by the distance between the center of the chair and the center of the display), but not for the exact viewing distances or potential head and upper body movements. This allowed us to validate the Virtual Chinrest in a more natural setting, with participants sitting in front of the computer as they would at home.

12 participants took part. We adopted a within-subjects experimental design with the seating distance and the display size as two factors. The seating distance had three levels, 43, 53, and 66 cm, and the display size had two levels of 13′′ and 23′′. Participants were instructed to complete the same experimental procedure as Exp. 1 in 6 (3 × 2) conditions.

**Figure 3:**
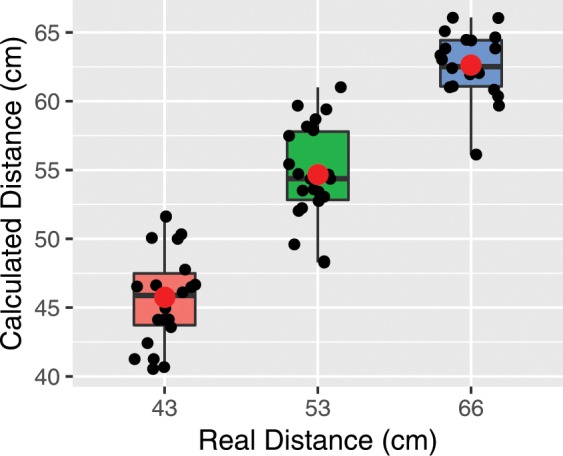
The box plot and the 12 individual viewing distances calculated using Virtual Chinrest in three distance conditions (43, 53, and 66 cm or 17, 21, and 26 inch) in Exp. 2. The red dots represent the calculated mean distance in each condition. The average absolute error is 3.25 cm (sd = 2.40 cm) across all three conditions.

Our results show that the Virtual Chinrest detects users’ seating distance (as a proxy for viewing distance) with an average absolute error of 3.25 cm (sd = 2.40 cm). Table 1 and Fig. 3 present the results of the different conditions: among the 3 distances, the viewing distance of 53 cm was predicted most accurately with an average absolute error of 2.88 cm (mean ± sem = 54.7 ± 0.76 cm). The viewing distance of 43 cm was predicted least accurately with an average absolute error of 3.46 cm (mean ± sem = 45.8 ± 0.74 cm). We found that the viewing distances were over-estimated by 1.4 cm when the larger display (23′′) was used and underestimated by 0.86 cm when the smaller display (13′′) was used. A paired t-test confirmed this difference is statistically significant (*t*_(31)_ = 4.56*, p* < 001). However, despite the small amounts of bias introduced by these different conditions, the overall accuracy was still very high.

**Table 1:** Calculated viewing distances of each condition using Virtual Chinrest in Exp. 2, a 3 × 2 within-subjects lab study.

Actual Distance (cm)	Estimated Distance Mean (Avg. Abs. Err)		
	13″	23″	*Average*
43	47.2 (4.6)	44.3 (2.4)	45.8 (3.5)
53	55.7 (4.2)	53.6 (1.6)	54.7 (2.9)
66	63.7 (2.4)	61.7 (4.4)	62.6 (3.4)

#### Horizontal blind spot location estimation

**Figure 4:**
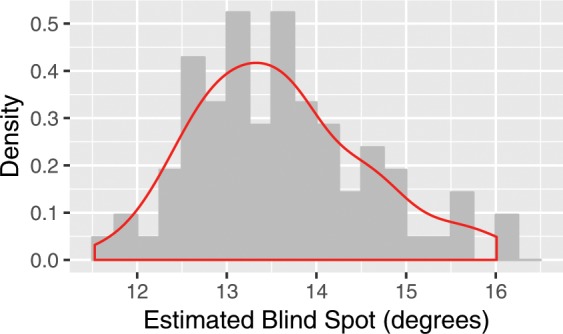
The distribution of the estimated horizontal blind spot entry point locations (mean = 13.59°, sd = 0.96°) of 30 participants, 85 experimental sessions from Exp. 1 and Exp. 2.

Both Exp. 1 and Exp. 2 in which we controlled for viewing distances or seating distances allowed us to calculate participants’ horizontal blind spot locations based on the data from the blind spot tasks. Combining the data from both experiments, the mean horizontal blind spot entry point location is 13.59° (min = 11.53°, max = 16.01°) with a SD of 0.96°. The distribution of the estimated blind spot locations is plotted in Fig. 4. Since the mean blind spot diameter is around 4.5°^22,25^, the center of the blind spots from our results is then 15.84° ± 0.96°, comparable to prior work, in which the blind spot locations are ranged between 14.33^◦^ and 15.52°^22,23,25^.

The discrepancy of the average blind spot locations from previous studies (e.g.^22,23^) and our own finding that the horizontal blind spot locations ranged between 11.53° and 16.01° may suggest that any minor inaccuracies in our viewing distance estimation was caused by variations across individuals’ blind spot locations.

### Online replication of a laboratory study on visual crowding using the virtual chinrest

In Exp. 1 and Exp. 2 we have demonstrated that the Virtual Chinrest is highly accurate in measuring the viewing distances, even in relatively uncontrolled settings with variable viewing distances, display sizes, and potential movements of the head and upper torso. This allows us to further examine whether we can use the Virtual Chinrest to conduct the type of studies that would typically rely on a physical chinrest, in an uncontrolled online environment. We aim to replicate classic findings from psychophysical experiments measuring visual crowding (e.g.^27–30^) — studies that require precise control over the retinal location of stimuli. Visual crowding is a phenomenon that occurs in peripheral vision where an observer’s ability to identify a target is greatly reduced when the target is flanked by nearby objects. Using the visual crowding paradigm, we can measure individual differences of visual crowding effects, i.e., how much distance between the target and flankers one needs to correctly identify the target. These individual differences in low-level visual processing have been related to high-level cognitive function such as reading ability^31,32^. Measuring an individual’s crowding effect requires being able to (a) present the target at the same eccentricity and (b) manipulate the distance between the target and flankers using the same units (i.e., in visual angle) across individuals. Thus, without knowing the viewing distance and the display size, it is impossible to measure an individual’s crowding effect.

**Figure 5:**
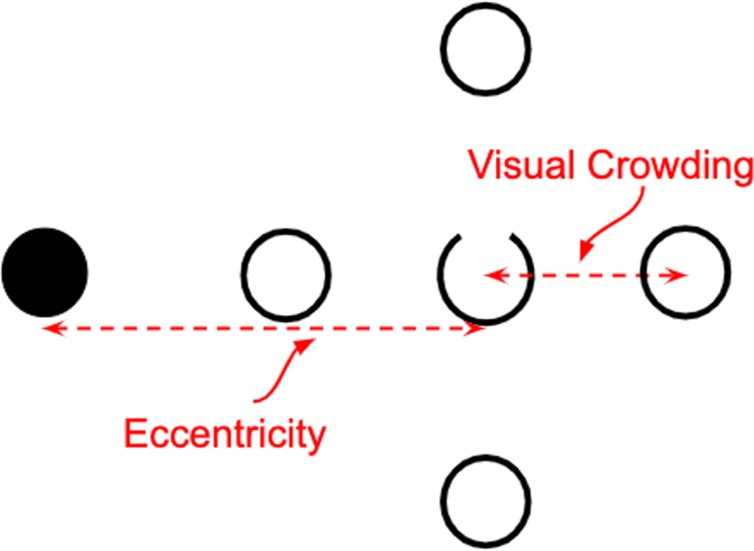
The main stimuli used in the visual crowding experiments (Exp. 3 and Exp. 4): After 500 msec of fixation on the central mark, crowding stimuli appeared at either the left or the right side of the display. The stimuli disappeared after 150 msec and participants reported the direction of the gap (up or down) using the keyboard.

We developed a version of the visual crowding experiment (see stimuli in Fig. 5) as a 10-minute online test that began with setting up the Virtual Chinrest (by asking participants to perform the card task and the blind spot test). Each participant was randomly assigned one target eccentricity, 4° or 6°. Participants were then presented instructions for the visual crowding experiment and asked to perform a practice session with 5 trials. The main experiment was split into two blocks and each block was followed by another blind spot task to determine whether participants have changed position.

#### Exp. 3. Validation of browser-based measurements of crowding

Our first goal was to ensure that our browser-based implementation of the visual crowding experiment can gather high quality data with and without a Virtual Chinrest. To do so, we conducted a within-subjects laboratory study in which 19 participants took the experiment (with the same target eccentricity assigned to each of them) in two conditions: (1) using a physical chinrest set to a viewing distance of 53 cm and (2) using the Virtual Chinrest on a laptop in their desired position (i.e., on the lap or on a desk) and desired distance. The latter condition was intended to simulate an *in-situ* environment that participants might find themselves in when participating in an online experiment. We compared an individual’s crowding effect between the two conditions.

**Figure 6:**
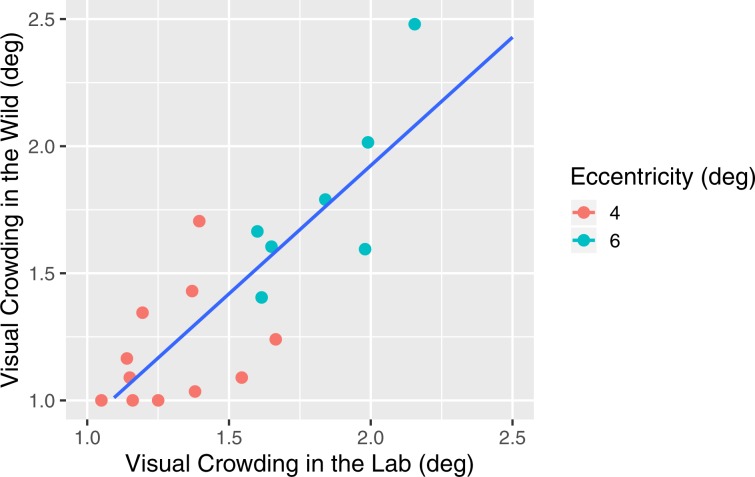
The visual crowding measures in Exp. 3 were significantly correlated (Pearson’s r = 0.86, p < 0.001, n = 18) in the controlled and uncontrolled laboratory settings where 18 participants successfully completed the visual crowding experiment both in the lab with a physical chinrest and using the Virtual Chinrest on a laptop in their desired position and distance. Visual crowding effects increased as the eccentricity of the target increased (mean = 1.228° at 4° and mean = 1.811° at 6°, *t*_*(*9.08)_ = *−*5_._122, *p* < 0.001 by Welch’s two sample t-test), confirming conventional eccentricity-dependent crowding effects.

Results of 18 participants show that individuals’ crowding effect measures are highly correlated in the controlled and uncontrolled laboratory setting (Pearson’s *r* = 0.86, *p* < 0.001, *n* = 18), suggesting that individual differences can be precisely reproduced using the Virtual Chinrest when not controlling for viewing distance and angle (we removed the data of one participant who did not correctly follow the instruction). Figure 6 presents each individual’s crowding effect in the two conditions, grouped by eccentricity. The results are aligned with previous findings that the crowding effect is linearly dependent on eccentricity^30,33–35^. A Welch's two sample t-test showed that the average crowding effect is 1.228° when eccentricity is 4°, which is significantly different from the average crowding effect of 1.811° when eccentricity is 6° (*t*_(9.08)_ = *−*5.122*, p* < 0.001).

#### Exp. 4. Visual crowding experiment in the wild

To evaluate whether we can replicate results from the visual crowding experiment in a truly uncontrolled environment, we conducted an online experiment on the volunteer-based experiment platform LabintheWild^12^. LabintheWild attracts participants from diverse demographic and geographic backgrounds^3,10–12^. Participants use a wide range of browsers, devices, and displays, and take experiments in a variety of situational lighting conditions and seating positions^3^. Our goal is to evaluate whether we can accurately replicate the visual crowding experiment despite this diversity.

**Figure 7:**
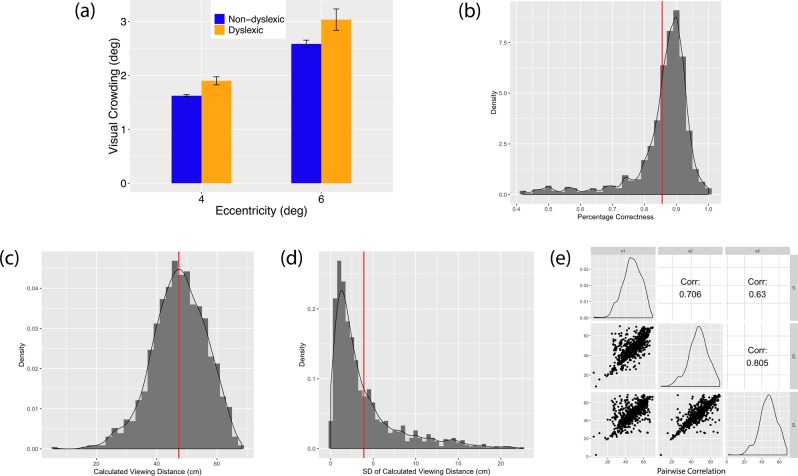
The results of Exp. 4 where 793 participants completed the visual crowding experiment implemented using Virtual Chinrest on LabintheWild. (**a**) The average visual crowding effects were significantly different between target eccentricity of 4° (mean = 1.61°) and 6° (mean = 2.66°), and between participants with (e = 4^*◦*^: 1.90°; e = 6°: 3.03^*◦*^) and without (e = 4°: 1.62°; e = 6°: 2.58°) dyslexia. Error bars represent standard error. (**b**) The distribution of the percentage correctness of the crowding experiment across all participants. The average (indicated by the red vertical line) is 85.56%. (**c**) The distribution of the viewing distances across all participants calculated by Virtual Chinrest. Our participants’ viewing distances were between 17.4 cm and 68.3 cm with mean = 47.3 cm and sd  = 8.9 cm. (**d**) The distribution of the within-subjects standard deviation (SD) of the viewing distances across all participants: the average is 3.9 cm (min = 0.003 cm, max = 22.7 cm). (**e**) The pairwise correlation of calculated viewing distances among three blind spot tasks at the beginning (s1), in the middle (s2) and at the end (s3) of the crowding experiment. The correlations of calculated viewing distances between s1 and s2, s2 and s3, s1 and s3 are 0.706, 0.805 and 0.630, respectively.

Our experiment results, based on the data of 793 participants, replicate the previously found positive correlation between crowding effect and eccentricity^30,33–35^. More precisely, we compared the crowding effect between two target eccentricities. The results showed that participants’ crowding effect increased as the eccentricity of the target increased from 4° (mean = 1.61°, sem = 0.02°) to 6° (mean = 2.66°, sem = 0.06°), and a non-parametric Mann-Whitney *U* test confirmed that the results are statistically significant (*W* = 60502, p < 0.001; Fig. 7a), confirming eccentricity-dependent crowding effects from previous studies^30,33–35^.

Since we had a large and diverse sample, we further tested whether and how other covariates might be predictive of visual crowding: We ran a linear mixed-effects regression model with visual crowding as the dependent variable and participant as a random variable. We included *age* and *age_squared* (i.e., the square of the variable *age*) as fixed effects. Other fixed effects were *eccentricity* (4° or 6°), *dyslexia* (1 or 0) and *gender*. As shown in Table 2, the eccentricity-dependent crowding effects held even when controlling for these other variables.

**Table 2:** The results of a quadratic mixed-effect model predicting visual crowding.

Variable	Est.	SE	t-value	Pr(>|t|)
(Intercept)	−0.38	−0.20	−1.88	=0.06.
Eccentricity [6°]	0.53	0.02	21.39	<0.001^∗∗∗^
Dyslexia [yes]	0.26	0.10	2.70	<0.005^∗∗^
Age_squared	0.004	0.001	2.68	<0.005^∗∗^
Age	−0.02	0.01	−1.77	=0.07
Gender	0.04	0.06	0.74	0.46 (n.s.)

Our results show that people who self-reported having been diagnosed with dyslexia (N=59, excluding 10 who reported having additional impairments) have significantly higher visual crowding than those without in both target eccentricity conditions, consistent with the findings of prior work^30,32,36^, although the relationship between dyslexia and visual crowding is highly debated^31,37–40^ (Fig. 7a). In addition, we found that visual crowding is roughly half of the eccentricity: the ratio of the crowding to the eccentricity is 0.40 (4°) to 0.44 (6°), following the Bouma’s law^41,42^ also conformed by other studies^32,43^. Age also significantly impacted visual crowding, confirming previous findings demonstrating increased visual crowding in aging populations^44–46^. Moreover, we find increased crowding in young children compared to adults. Thus, there is a quadratic relationship between crowding and age, and individuals with dyslexia (on average) display increased crowding across the sampled age range.

The average accuracy of the crowding experiment (50 trials) across all participants was 85.56% (median = 88%, max = 100%, min = 42%; Fig. 7b). This is aligned with the 79.4% correction rate of the 1-up 3-down staircase procedure, which demonstrates that we obtained reliable and accurate data the same as observed in our laboratory study or in prior work^47^.

Our experiment included three blind spot tests at the beginning, in the middle, and at the end of the study to evaluate whether and how much participants move and whether it is sufficient to only assess their viewing distance once for a 10-minute online study. In our online experiment, participants varied in their viewing distance between 17.4 cm and 68.3 cm (mean = 47.3 cm, sd = 8.9 cm, see Fig. 7c). As shown in Fig. 7d, the average within-subjects standard deviation of estimated viewing distances (across the three blind spot tests) is 3.9 cm (min = 0.003 cm, max = 22.7 cm). Estimated by a one-way random effects model with absolute agreement, the intra-class correlation of a participant’s estimated viewing distances before, during and after the crowding experiment is *ρ* = 0.88 (see Fig. 7e for pairwise correlations). This suggests that different participants vary substantially in their viewing distance (underlining the need for a Virtual Chinrest), but participants do not move much over the course of a 10-minute online experiment. We found no substantial difference in visual crowding between people who moved more and less, and therefore, assessing the viewing distance once at the beginning of an experiment may be sufficient for most participants.

## Discussion

This paper introduced the Virtual Chinrest, a novel method that allows estimating participants’ viewing distances, and thus, calibrating the size and location of stimuli in online experiments. We validated our method in two laboratory studies in which we varied the viewing distance and display size, showing that the Virtual Chinrest estimates participants’ viewing distances with an average absolute error of 3.25 cm – a negligible error given an average viewing distance of 53 cm. Using the Virtual Chinrest in an online environment with 1153 participants, we were able to replicate and extend the results of a laboratory study on visual crowding, which requires particularly tight control of viewing distance and angle. More specifically, we replicated three prior findings: (1) the positive correlation between the crowding effect and eccentricity in^30,33–35^, (2) the finding that participants with dyslexia experience higher visual crowding than those without dyslexia^30,32,36^, and (3) the increase in visual crowding that occurs with aging^44–46^. Moreover, we extended these results by showing that there is a quadratic relationship between age and visual crowding. Our findings pave the way for laboratory studies requiring a physical chinrest to be conducted online, enabling psychophysical studies with larger and more diverse participant samples than previously possible.

The Virtual Chinrest is not necessary for all types of psychophysical online experiments. For example, prior work has successfully replicated visual perception experiments on proportional judgments of spatial encodings and luminance contrast, and investigated the effects of chart size and gridline spacing for optimized parameters for web-based display via online experiments, without controlling for viewing distance, display size or resolution^2,21^. These prior experiments followed a within-subjects design, did not require cross-device comparisons, and the results are not sensitive to changes in visual degrees.

However, there are two main types of visual perception studies that are unlikely to replicate if conducted without controlling for participants’ viewing distance: (1) between-subjects studies that compare specific metrics across participants, because they require a consistent measurement across various environments and devices, and (2) any study that requires visual stimuli sizes to be the same across participants. Visual crowding is a prime example, where thresholds are expressed in units of visual angle. For these types of study designs, each participant’s viewing distance derived from the Virtual Chinrest can be used for adapting the size of the stimuli and/or as a control variable in the analysis.

Our results show that after instructing online participants to keep their position throughout the experiment, they indeed move very little – on average, participants viewing distance changed by 3.9 cm. This suggests that for 10-minute experiments, asking participants to take the 30 seconds to set up the Virtual Chinrest once at the beginning of the experiment may be enough. For longer experiments, we suggest assigning the Virtual Chinrest tasks multiple times throughout the experiments to adjust the stimuli correspondingly.

In summary, we developed the Virtual Chinrest to measure a person’s viewing distance through the web browser, enabling large-scale psychophysical experiments of visual perception to be conducted online. Our method makes it possible to automatically adjust the stimulus configurations according to each participant’s viewing distance, producing reliable results for visual perception studies that are sensitive to display parameters. We hope that our method will enable researchers to leverage the power of studies with large and diverse online samples, which often have greater external validity, can detect smaller effects, and have a higher probability of finding similarities and differences between populations than traditional laboratory studies.

## Methods

### Lab study

In Exp.1, 19 participants completed the Virtual Chinrest experiment (consisting of the card task and blind spot test) using a physical chinrest in a psychophysical experiment room. Each participant completed the experiment once. In Exp. 2, 12 new participants performed the same experiment, but without a physical chinrest. The 2 × 3 within-subjects experiments used two different-sized screens (13′′ and 23′′) and three seating distances: 43, 53, and 66 cm (17, 21, and 26 inch). We chose 53 cm because it was the distance used in the original laboratory study, and we chose 43 cm and 66 cm because the International Organization for Standardization (ISO) guidelines suggest distances between 40 cm and 75 cm are reasonable choices^48^. The 6 conditions were counterbalanced across participants.

#### Setup

Participants completed the experiment in a room with controlled artificial lighting. Exp. 1 was conducted using a 24′′ monitor (Model: LG 24GM77-B) with a resolution of 1920 × 1080. The viewing distance was set to 53 cm (21 inch). Exp. 2 was conducted with two monitors: a 13′′ Macbook Pro with a resolution of 2560 × 1600 pixels and a 23′′ monitor (Model: HP Compaq LA2306x) with a resolution of 1920 × 1080 pixels. To perform the card task, participants were provided a card of size 85.60 × 53.98 mm (a standard credit card size) in both experiments. The setup remained the same throughout the entire experiments.

#### Procedure

Both experiments asked participants at the beginning to assume a comfortable position and to keep this position throughout the experiment. The experiment started with an informed consent form, a demographic questionnaire, followed by the Virtual Chinrest experiment consisting of two tasks. During the blind spot task (Fig. 1b), participants were instructed to press the spacebar as soon as the red dot disappears from their left eyesight and repeat this process 5 times so that later we calculated the viewing distance by taking the average of the results. The participants in Exp. 1 (with chinrest) only completed the tasks once while participants in Exp. 2 (without chinrest) completed the tasks in all 6 conditions. Completion of the experiment in each condition took approximately 4 minutes. All experimental sessions were approved by the University of Washington Institutional Review Board and performed in accordance with the relevant guidelines and regulations.

#### Participants

A total of 19 participants and another 12 distinct participants completed the experiments in Exp.1 and Exp. 2, respectively. All of the participants were recruited from a local university, and all self-reported having normal or corrected-to-normal vision. Written informed consent was obtained from all participants.

#### Analysis

For the analysis of Exp. 2, we removed one participant who did not successfully complete the entire experiment. We also removed one data point of another participant who did not correctly complete the card task in the condition of [66 cm, 13′′].

### Online experiment

The online experiment was launched on the volunteer-based online experiment platform LabintheWild and advertised with the slogan “How accurate is your peripheral vision?” on the site itself as well as on social media.

#### Experimental design

During each experimental session, we first presented the Virtual Chinrest experiment and used the results to calculate individual’s viewing distance and to calibrate the stimuli’s size and locations. Instead of creating stimuli (demonstrated in Fig. 5) using MATLAB, we created the stimuli as SVG on HTMLs and manipulated the stimuli using JavaScript. All the elements were created in a container with a width of 900 pixels on the webpage. In the blind spot test, the dot was drawn in red with a diameter of 30 pixels, and the fixation square was drawn in black with a side length of 30 pixels (Fig. 1b). Replicating the original crowding study^30^ in the unit of visual degrees, stimuli comprised four flankers — open circles with 1° diameter and a target — an open circle with a gap (target; an arc with reflex central angle of 330°). All stimuli were black and displayed on a white background (Fig. 5). Two conditions of target eccentricity (the center-to-center distance between the fixation mark at the center of the webpage and the target) were 4° and 6°. In each crowding experiment session, each participant was randomly assigned one target eccentricity, and the target eccentricity was fixed with the starting target-flanker distance being set as 1.3 times greater than half the eccentricity (3.9° for 6° eccentricity; 2.6° for 4° eccentricity).

During each crowding experiment session, the subsequent target-flanker distances (25 trials/steps in total) were controlled by the 1-up 3-down staircase procedure implemented in JavaScript [https://github.com/hadrienj/StaircaseJS]. On a given trial, the fixation mark was displayed first and remained on the webpage for the entire session. After 500 ms of fixation onset, the stimuli were displayed either to the left or the right of the fixation for 150 ms. Only the fixation remained on the webpage until the participant submitted a response by using the arrow keys on the keyboard to indicate the direction (up or down) of the target gap. No feedback was provided during the experiment. There was a 500 ms blank between a participant’s response and the beginning of the next trial.

The visual crowding, defined as the minimal center-to-center distance between a target and the flankers (in degrees), was used to quantify the crowding effects when participants could report the target identity at certain accuracy. Since we are using a 1-up 3-down staircase procedure, participants should be able to correctly report the target identity 79.4% of times.

#### Procedures

The experiment began with a brief overview of the study, an informed consent form approved by the University of Washington Institutional Review Board, and a voluntary demographic questionnaire, followed by the card task and the blind spot test with 5 trials to calculate participants’ viewing distances. Participants were then presented the instruction of the crowding tasks and a practice session with 5 trials.

The main experiment was split into two blocks (two independent staircases, 25 trials each), and each was followed by another blind spot task with 3 trials. After the last blind spot test, participants were then given the opportunity to report on any technical difficulties, and to provide any other general comments or questions. The final page showed their personalized “crowding effect” in comparison to others. The entire study took 10–12 minutes to complete. All experimental sessions were approved by the University of Washington Institutional Review Board and performed in accordance with the relevant guidelines and regulations.

#### Participants

The experiment was deployed online for 15 months and completed 1198 times. We excluded 45 participants who self-reported participating more than once. Our analysis therefore reports on the data of 1153 participants. Informed consent was obtained from all participants.

Participants were between 7–71 years old (mean = 26.3, sd = 12.4) and 50.2% were female. 229 participants reported to have cognitive impairments, including dyslexia, learning disability, reading difficulties and Attention Deficit Disorder (ADD). 69 (6.0%) of all participants reported to have dyslexia. The plurality of participants (32.9%) reported having completed college, 21.3% completed graduate school, and 19.8% completed high school. The remaining participants were enrolled in professional schools, pre-high school, or unspecified.

#### Analysis

We deployed the online study in two stages, where we added more granular data log at the second stage, such as the percentage correctness of the experiment and the results of each individual trial. Therefore, the analysis of visual crowding effects (Fig. 7a,b) was performed on the data of 793 participants from the second stage, the results in Table 2 was based on a subset of 570 participants who have explicitly reported whether they have dyslexia and/or other related impairments, while the results of the viewing distances from the three blind (Fig. 7c–e) spot tests were reported from all 1153 participants.

We checked for data normality by both the visual inspection of histograms and the Shapiro-Wilk normality tests before each analysis. We then conducted parametric (e.g. the Welch’s two sample t-test) and non-parametric (e.g. Mann-Whitney *U* test) analysis accordingly. In the linear mixed-effects regression models, t-tests (p-values) were calculated using Satterthwaite approximations for the degrees of freedom.

The data analysis of all four experiments was performed in R, with the help of multiple packages^49–52^.

## Data Availability

We make available the Virtual Chinrest as a JavaScript library, all data from our lab studies, online study, and the R-code for analysis at https://github.com/QishengLi/virtual_chinrest/.
